# The Role of Autoimmunity in the Pathogenesis of Sudden Sensorineural Hearing Loss

**DOI:** 10.1155/2018/7691473

**Published:** 2018-06-13

**Authors:** Guangfei Li, Dan You, Jiaoyao Ma, Wen Li, Huawei Li, Shan Sun

**Affiliations:** ^1^Key Laboratory of Hearing Medicine of NHFPC, ENT Institute and Otorhinolaryngology Department, Shanghai Engineering Research Centre of Cochlear Implant, Affiliated Eye and ENT Hospital, State Key Laboratory of Medical Neurobiology, Fudan University, Shanghai 200031, China; ^2^Institutes of Biomedical Sciences and The Institutes of Brain Science and the Collaborative Innovation Center for Brain Science, Fudan University, Shanghai 200032, China

## Abstract

Sudden sensorineural hearing loss (SSHL) is a clinically common acute symptom in otolaryngology. Although the incidence of SSHL has increased around the world in recent years, the etiology of the disease is still unclear. It has been reported that infections, ototoxic drugs, membrane labyrinth rupture, carcinomas, circulatory system diseases, autoimmune diseases, brain lesions, mental diseases, congenital or inherited diseases, and so on, are all risk factors for SSHL. Here, we discuss the autoimmune mechanisms behind SSHL, which might be induced by type II–IV allergic reactions. We also introduce the main immunosuppressive medications that have been used to treat SSHL, which will help us to identify potential targets for immune therapy.

## 1. Introduction

Sudden sensorineural hearing loss (SSHL) or “idiopathic sudden sensorineural hearing loss” refers to the sudden, unexplained hearing loss of more than 30 dB across all frequencies. The main clinical symptom is hearing loss, sometimes accompanied by tinnitus, ear blockage, dizziness, nausea, and/or vomiting. The pathogenesis of SSHL involves complex systemic or regional symptoms, and there are as yet no effective treatments. Here, we review our current understanding of SSHL and inner ear structures and cells as the fundamental platform for immune surveillance and responsiveness in hearing loss, and we present a summary of SSHL that results from immune system dysfunction. We think that immune-modulating medications support the clinical findings and suggest some potential targets for therapy in clinics.

## 2. The Evidence for Autoimmunity in SSHL

The inner ear and brain are traditionally viewed as being immune privileged because there is a blood-labyrinthine barrier that acts in a similar manner as the blood-brain barrier, and only a few macrophages are present in these organs [1]. However, a large number of experimental and clinical cases of SSHL have been identified in which SSHL is a symptom associated with other autoimmune diseases or is the primary symptom of spontaneous systemic autoimmune diseases such as autoimmune hepatitis [2], sympathetic neural hyperalgesia edema syndrome [3], Cogan's syndrome [4, 5], systemic lupus erythematosus [6, 7], multiple sclerosis [8–10], rheumatoid arthritis [11], nodular polyarteritis [12], Crohn's disease [13], and so on. Increasing experimental evidence suggesting an autoimmune component in the pathology of SSHL has emerged since 1979 when McCabe first identified 18 patients with autoimmune-associated SSHL who were effectively medicated with glucocorticoid and vincristine [14]. The presence of antibodies against the inner ear 68 kDa antigen and the recovery of hearing after immunosuppressive therapy have further confirmed the immune-mediated mechanism of hearing loss [15–18]. Immunohistochemistry and other techniques have been used to show that immune cells, including lymphocytes, leukocytes, and macrophages, are present in the inner ear as well as to analyze the interactions between these immune cells [19–22]. The following inner ear antigens (see Table 1) are considered to be the main targets of harmful antibodies: 68 kDa protein [23, 24], 30 kDa protein (also called myelin protein zero (P0)) [25], collagen type II [26, 27], tubulin [28], cochlin [29, 30], and inner ear supporting cell antigen [23]. Moscicki et al. have confirmed the clinical relationship between idiopathic SSHL and anti-68 kDa protein antibodies in patient serum [22, 31]. Furthermore, Billings et al. [24] and Bloch et al. [16, 17] have confirmed that the 68 kDa protein is heat shock protein 70 (HSP-70). These studies have provided a basis for the diagnosis and treatment of autoimmune-related SSHL.

## 3. The Immune Response in the Inner Ear

The immune system plays an important role in protecting the inner ear from damage caused by bacteria, viruses, and other pathogenic microorganisms. However, in the pathogenesis of autoimmune hearing loss, the immune system itself damages the inner ear. Although the exact mechanism of its pathogenesis is not yet fully understood, studies in patients with SSHL and in experimental animal models have identified a number of factors that are involved in autoimmune SSHL. The immune response in the inner ear relies on cytokines, especially IL-1*β* [32, 33], IL-2, and TNF-*α* [34], that play important roles in regulating the immune response of the inner ear. Some inflammatory cells in the inner ear are also involved, including macrophages (or microglia-like cells), T lymphocytes, and leukocytes. Our previous work has demonstrated that the ototoxicity of neomycin (an aminoglycoside antibiotic) is mediated through the activation of microglia-like cells that release proinflammatory cytokines that cause damage to the hair cells of the inner ear [35, 36].

### 3.1. The Physiological Immune Defense in the Inner Ear

**Figure 1:**
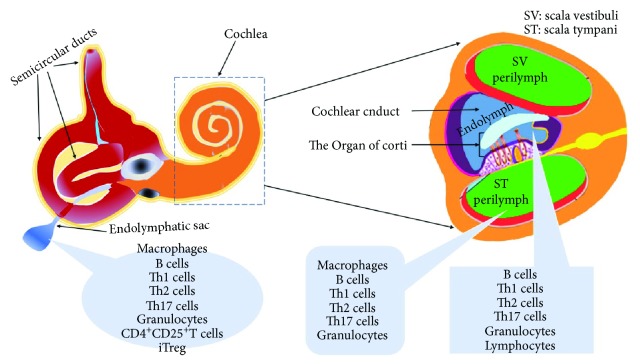
The distribution of immune cells in the inner ear when the immune response is initiated.

The inner ear is fully capable of initiating an immune response to the invasion of external antigens. Previous studies have shown that the lymphatic sac contains several of the immunological components of the immune response and is the primary site of the immune response [37, 38]. The antigens in the inner ear are often used as targets for such immune responses. Recognition of these antigens by the inner ear's innate immune cells (neutrophils, macrophages, dendritic cells, etc.; see Table 2 and Figure 1) stimulates the release of IL-1*β*, which in turn triggers a series of adaptive immune responses. The cytokines that are released as part of these responses then recruit lymphocytes from the circulatory system into the inner ear where they cause irreversible tissue damage [39].

### 3.2. Pathological Immunity in the Inner Ear

No lymphocytes are present in the normal endolymphatic sac, and there is no evidence that the lymphocytes present in the cochlea during the immune response are derived from the endolymphatic sac; thus, they must originate mainly from the peripheral circulatory system [40]. Lymphocytes in the circulatory system are predominantly migrating from the spiral vessels and their branches [41]. When they reach the other organs of the body, they initiate the process of antigen absorption, presentation, and degradation. IL-1 plays an important role in regulating the innate immune response, and it acts as an agonist of resting helper T cells and B cells. The helper T cells, once activated by IL-1, will produce IL-2. The secretion of IL-2 results in pluripotent stem cells differentiating into helper T cells, cytotoxic T cells, and suppressor T cells. IL-2 also assists helper T cells in activating B lymphocytes and might play an important role in regulating the immune response in the inner ear [42].

IL-1*β* and TNF-*α* are involved in the initiation and amplification of immune responses. IL-1*β* is mainly expressed in the fibroblasts of the spiral ligament in the case of nonspecific trauma such as surgery or acoustic neuroma, while TNF-*α* is mainly expressed in infiltrating circulating inflammatory cells or innate immune cells in the endolymphatic sac under the stimulation of external antigens. The release of TNF-*α* in animal models is a part of the adaptive immune response. When an antigen is injected into the mouse inner ear, both IL-1*β* and TNF-*α* are secreted and a normal immune response occurs. However, when the antigen flows from the cerebrospinal fluid to the inner ear and the inner ear is not traumatized, only TNF-*α* is secreted and only a very weak immune response is initiated. It is worth noting that damage to the cochlea alone can also lead to a slight immune response [43]. These results all show that the nonspecific and specific components of the immune response act synergistically in the inner ear so as to maximize the effect of the immune response.

Therefore, if the cochlea is damaged or antigens are injected into the cochlea (or a patient with an autoimmune disease has immune cells directly attacking the inner ear antigen), both nonspecific and specific immune responses are activated simultaneously, and these can result in simultaneous IL-1*β* and TNF-*α* production that amplifies the inflammatory effect and then leads to extensive damage to the inner ear tissue. Animal model experiments have confirmed that the innate immune response predominates in the inner ear until the regulatory immune response produces enough of an inflammatory response to cause damage to the inner ear. Therefore, when innate and specific immune responses are activated at the same time, it might be possible to avoid excessive immune responses by downregulating or inhibiting specific immune responses, particularly those that inhibit the effects of TNF-*α*.

## 4. The Immune Pathogenesis of Hearing Loss

**Figure 2:**
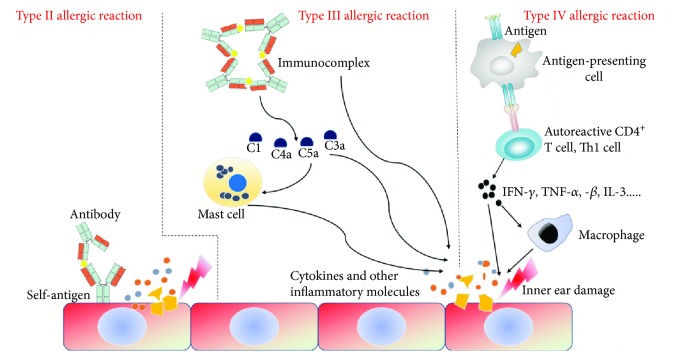
The mechanisms of inner ear damage by the type II–IV allergic reactions.

Although it is known that immune responses in the inner ear can lead to tissue damage, the exact mechanism behind the injury process remains unclear, and thus we can use other autoimmune diseases as a reference to understand such injury processes in the inner ear. In general, immune response damage is mediated by both humoral and cellular immunity, and autoimmune damage can be classified as type I allergic reactions to type IV allergic reactions. Type I allergic reactions (immediate-type allergic reactions) are mainly caused by the interactions of an antigen with an antibody (usually IgE) on the surface of immune cells that activate the cells and causes them to release active mediators such as histamine and serotonin to induce a rapid immune response. Type II allergic reactions (cytotoxic allergies) are mediated by IgG or IgM, and when the antibody binds to the antigen on the foreign cell surface, the cells are destroyed due to the action of the complement system, phagocytes, or nature killer cells. Type III allergic reactions (immune complex allergies) are caused by the deposition of medium-sized soluble antigen-antibody complexes into capillary walls or tissues, which activates the complement system or leads to the recruitment of leukocytes. Type IV allergic reactions (delayed-type allergies) cause tissue injury that is mediated by T cells. Type I allergic reactions are not associated with autoimmune hearing loss, but types II–IV (see Figure 2) have been shown to be potential mechanisms that lead to inner ear damage in autoimmune SSHL [44], and these are described in the following sections.

### 4.1. Type II Allergic Reactions (Cytotoxic Allergies)

Type II cytotoxic antibody-mediated damage can be confirmed from previous animal studies and clinical studies. Harris [45, 46] injected KLH protein, a metalloprotein extracted from snails, into susceptible guinea pigs. The exposure to KLH resulted in the production of anti-KLH antibodies. Subsequent injection of bovine inner ear antigen into guinea pigs resulted in hearing loss, and circulating antibodies specific to bovine inner ear antigen were found in the serum and perilymph. In patients with SSHL, analysis of antibodies in the inner ear using Western blotting revealed that there were IgG antibodies against the inner ear-specific proteins cochlin and *β*-tectorin and the nonspecific protein HSP-70. This study revealed that the direct antibody response to inner ear proteins can lead to SSHL and that such antibodies can be used as a marker for disease diagnosis. Using antigen-specific Western blot analysis of patient and healthy sera, it was found that anti-cochlin IgG antibodies were more prevalent in patients with idiopathic sensorineural hearing loss than anti-*β*-tectorin-specific IgG antibodies, whereas anti-HSP-70 IgG antibodies were more common than anti-cochlin IgG antibodies and anti-*β*-tectorin-specific IgG antibodies in all of the patients [47]. These animal experiments and clinical studies have provided compelling evidence that in at least some patients with SSHL the pathology is due to antibody-mediated tissue damage in the inner ear.

### 4.2. Type III Allergic Reactions (Immune Complex Allergies)

Type III immunocomplex-mediated hearing loss mechanisms have been identified in animal models of SSHL. Especially in C3H/lpr autoimmune mice with progressive hearing loss, one can find deposition of immunocomplexes in the vascular stria [48], and the deposition of IgM and IgG immunocomplexes can be seen in NZB/kl mice that have high incidences of hearing loss [49]. Trune et al. found the presence of DNA antibodies in the inner ear of MRL/lpr mice, and such anti-DNA or DNA-anti-DNA antibody immunocomplexes result in the destruction of endothelial cell integrity that affects the function of the blood-labyrinthine barrier resulting in SSHL [50]. Although the transfer of findings from animal models to patients is still speculative, the link between systemic autoimmune diseases and SSHL can provide additional evidence for the existence of such a mechanism. Many clinical cases have described hearing loss patients with associated systemic autoimmune disorders, and many of these systemic autoimmune diseases have been confirmed as type III allergic reactions with immunocomplex deposition resulting in tissue damage. For example, a 19-year-old girl with SSHL and mouth ulcers and bleeding under the nails was diagnosed with systemic lupus erythematosus. The histological sections revealed deposition of IgG, C3, C1q, and IgM immunocomplexes, obstruction of the vasculature of the inner ear by the formation of microthrombi, and damage to the organization of the inner ear. This patient's hearing recovered significantly with the use of methylprednisolone and other hormones [6]. Systemic autoimmune diseases such as multiple sclerosis, rheumatoid arthritis, and nodular arteriosclerosis are all associated with SSHL and are believed to form circulating immunocomplexes that deposit in the inner ear's vascular tissue thus causing SSHL.

### 4.3. Type IV Allergic Reactions (Delayed-Type Allergies): Autoreactive T Cell-Mediated Inflammatory Lesions

SSHL caused by type IV allergic reactions can be observed in animal models. Gloddek et al. used radioactive isotopes of chromium to label lymphocytes in susceptible experimental animals and demonstrated that lymphocytes migrate to the inner ear in response to antigenic stimulation and that infiltrated lymphocytes are found in the basal membrane and in the vestibule of the cochlea [51]. Hearing loss at all frequencies in the auditory brainstem response of mice was observed 5 weeks after immunization with the inner ear-specific protein cochlin 131–150 or *β*-tectorin in SWXJ mice. Each of the tested peptides activated Th1-like CD4^+^ T cells with proinflammatory effects as observed by flow cytometry analysis, and after 6 weeks of selective transfer of peptide-activated CD4^+^ T cells to unimmunized SWXJ mice, the auditory brainstem response threshold was significantly increased. This indicated that T cell-mediated tissue damage can lead to the development of autoimmune hearing loss, and immunocytochemistry analysis showed that the infiltration of leukocytes in the inner ear was associated with the observed hearing loss [52]. Billings also immunized SWXJ mice with cochlin 131–150 and confirmed that CD45^+^ T cells infiltrate the cochlea and cause autoimmune SSHL [53]. Zhou et al. used the inner ear autoantigen *β*-tubulin to create a mouse model of experimental spontaneous immune hearing loss. They showed that the response to *β*-tubulin involves CD4^+^ T cells producing *γ*-interferon, whereas T cell-mediated experimental autoimmune hearing loss is primarily caused by the induction of *β*-tubulin-activated CD4^+^ T cells in neonatal BALB/c mice and increased auditory brainstem responses were seen in mice in which these cells were activated. Furthermore, a significant decrease in CD4^+^/CD25^+^/Foxp3^+^ regulatory T cells was observed in mice immunized with *β*-tubulin, which inhibited the proliferation of effector CD4^+^/CD25^−^ T cells [54]. Xia et al. used flow cytometry to analyze the clinical T cell subtypes in 17 patients with autoimmune sensorineural hearing loss, 16 patients with noise-induced hearing loss, and 100 individuals with normal hearing. There was no significant difference in the T cell subtypes among the three groups, except that the proportion of CD4^+^ T cells in the patients with sensorineural hearing loss increased and the function of CD4^+^/CD25^+^ regulatory T cells was absent [55]. The above experimental animal models and clinical cases have confirmed that autoimmune hearing loss can be caused by cytotoxic T cell-mediated organ-specific autoimmune disorders of the inner ear.

## 5. Immunosuppressive Therapy for SSHL

Glucocorticoids have remained the main stay of treatment over the past four decades since McCabe [14] first treated SSHL with glucocorticoids, and the symptoms of patients were improved significantly. Owing to the systemic side effects of long-term treatment with glucocorticoids, other therapeutic methods also have been investigated. Ruckenstein et al. [56] and Trune et al. [57] used MRL/lpr mice to show that prednisolone can protect against hearing loss. In addition, Satoh et al. [58] and Wang et al. [59] used etanercept, a TNF-*α* antagonist, to treat SSHL and showed that it can reduce inflammation in the inner ear and prevent hearing loss. Clinically, Xenellis et al. [60] have shown that the intratympanic injection of steroids is a safe and effective method for SSHL treatment, and Haynes et al. [61] have shown that intratympanic injection of dexamethasone can also improve hearing in SSHL patients when systemic medications fail. Furthermore, Battaglia et al. [62] used a combination therapy of intratympanic dexamethasone with high-dose prednisone taper for SSHL and showed that the patients receiving the combination therapy had significant improvements in speech-discrimination score and pure-tone average and recovered their hearing quickly. More recently, azathioprine has been confirmed to maintain the hearing threshold, decrease the risk of relapse, and slow down the rate at which patients relapse [63].

The evidence to date suggests that autoimmune SSHL is mainly mediated by autoantibodies or T cells or by both. As autoimmune reactions are increasingly considered to be a cause of SSHL, animal models and clinical trials have shown that autoimmune processes cause damage to the inner ear through various mechanisms. Humoral immunity and cellular immune-mediated autoimmune damage have both been shown to play a role in the pathogenesis of autoimmune hearing loss. Although the precise diagnosis of autoimmune SSHL is still difficult, the response to immunosuppressive therapy is generally positive for these patients. Therefore, the immune mechanism of SSHL needs further study in order to identify specific antigens of the inner ear and specific diagnostic markers that can provide a more accurate and timely diagnosis and contribute to a more effective treatment plan.

## Figures and Tables

**Table 1:** The main autoimmune target antigens in the inner ear.

Inner ear antigen	Distribution	References
Collagen type II	In the subepithelial layer of the endolymphatic duct and spiral ligament.	[26, 64–68]
HSP-70	Hair cells and supporting cells.	[16, 17, 69–73]
*β*-Tubulin	Hair cells, supporting cells, the spiral ligament of the stria vascularis, and the spiral ganglion.	[54, 74–78]
Cochlin	In the regions of the fibrocytes of the spiral limbus and the spiral ligament and in the cochlear and vestibular labyrinth.	[52, 53, 79–82]
Beta-tectorin	Hair cells in the basal region of the hair bundle lying over the apical surface of the auditory epithelium, in the basilar papilla, in the clear cells and the cuboidal cells, and in the striolar region of the lagena macula.	[52, 53, 77, 83, 84]
Kresge Hearing Research Institute-3 (KHRI-3)	This is a protein specific to the inner ear and is expressed in the saccular wall cells and transitional epithelial cells in the utricle and ampules, by cells in the endolymphatic sac, and by supporting cells.	[85, 86]

**Table 2:** The innate immune cells and adaptive immune cells in the inner ear.

Immune cells		Function		Distribution	References
Innate immune cells	Macrophages	Promote the proliferation of T cells and B cells; Process and present antigens and participate in the regulation of adaptive immune responses; Phagocytosis and digestion of pathogenic microorganisms; Mediate and promote inflammatory responses		Endolymphatic sac and subepithelial and endoluminal space and the scala tympani and scala vestibuli.	[37, 38, 87, 88]
	Granulocytes	Anti-inflammatory, release of some inflammatory factors.		In the scala tympani, in the scala vestibuli from the basal turn to the apex, and in the modiolus.	[88–90]

Adaptive immune cells	B cells	Antigen identification and presentation. Activated B cells differentiate into plasma cells that secrete antibodies.		Peripheral circulation system, infiltrating into the endolymph and perilymph of the scala tympani and the scala vestibule and the endolymphatic sac.	[38, 91]
	Helper T cells	Th1 cells	Mediate cellular immunity; Secrete some cytokines, such as IFN-*γ*.	From the peripheral circulation system, infiltrate into the scala tympani, the scala vestibuli, and the perisaccular connective tissue of the endolymphatic sac and the modiolar vessels.	[20, 21, 52, 54, 82, 87, 91, 92]
		Th2 cells	Mediate humoral immunity; Secrete cytokines IL-4, IL-5, and IL-13.		
		Th17 cells	Secrete inflammatory cytokines IL-17 and IL-22; act as inflammation-initiating cells.		
	Suppressor T cells	CD4^+^/CD25^+^ Th cells	Negative regulator of immune response; CD4^+^CD25^+^ regulatory T cells play an immunosuppressive function in the periphery; iTregs can secrete the suppressive cytokines IL-10 and TGF-*β*.	From the circulation, infiltrate into the modiolus, the scala tympani, and the perisaccular connective tissue of the endolymphatic sac.	[55, 87, 92, 93]
		Antigen-specific regulatory T cells (inducible regulatory T cells (iTregs))			
	Lymphocytes	Anti-inflammatory		In the scala tympani, in all turns of the cochlea, and in smaller numbers in the scala vestibuli.	[51]
